# Identification of lncRNAs associated with uterine corpus endometrial cancer prognosis based on the competing endogenous RNA network

**DOI:** 10.7150/ijms.87430

**Published:** 2023-09-25

**Authors:** Liangxiao Wang, Xianwei Su, Liangyu Wang, Jianbo Luo, Zhiqiang Xiong, Geoffrey Ho Duen Leung, Jingye Zhou, Guang Yang, Li Zhai, Xi Zhang, Qiang Liu, Gang Lu, Yuming Wang

**Affiliations:** 1 Department of Clinical Laboratory, Yunnan Cancer Hospital, The Third Affiliated Hospital of Kunming Medical University, Kunming, 650106, Yunnan, China.; 2 Research and Development Unit, Shenzhen GenDo Medical Technology Co., Ltd., Dapeng, Shenzhen, 518000, China.; 3 Qujing Medical College, Qujing, 655000, Yunnan, China.; 4 SDIVF R&D Centre, 209,12W, HKSTP, Shatin, Hong Kong, China.; 5 Department of Sports Medicine, Qujing First People's Hospital, 650500, Yunnan, China.; 6 CUHK-SDU Joint Laboratory on Reproductive Genetics, School of Biomedical Sciences, The Chinese University of Hong Kong, Hong Kong SAR, China.; 7 Department of Clinical Laboratory, Yunnan Molecular Diagnostic Center, The Second Affiliated Hospital of Kunming Medical University, Kunming, 650500, Yunnan, China.

**Keywords:** lncRNAs, uterine corpus endometrial cancer, ceRNA network, diagnosis

## Abstract

Uterine Corpus Endometrial Carcinoma (UCEC) is one of the major malignant tumors of the female reproductive system. However, there are limitations in the currently available diagnostic approaches for UCEC. Long non-coding RNAs (lncRNAs) play important roles in regulating biological processes as competitive endogenous RNA (ceRNA) in tumors. To study the potential of lncRNAs as non-invasive diagnostic tumor markers, RNA-sequencing dataset of UCEC patients from The Cancer Genome Atlas was used to identify differentially expressed genes. A lncRNA-miRNA-mRNA ceRNA network was constructed by differentially expressed lncRNAs, miRNAs and miRNAs. Pathway enrichment and functional analysis for the mRNAs in the constructed ceRNA network provide the direction of future research for UCEC by demonstrating the most affected processes and pathways. Seven potential lncRNA biomarkers (C20orf56, LOC100144604, LOC100190940, LOC151534, LOC727677, FLJ35390, LOC158572) were validated in UCEC patients by quantitative real-time PCR. Notably, LOC100190940 and LOC158572 were identified as novel RNA molecules with unknown functions. Receiver operating characteristic (ROC) curve analysis demonstrated that the combined 7 lncRNAs had a high diagnostic value for UCEC patients with area under curve (AUC) of 0.941 (95% CI: 0.875-0.947). Our study highlights the potential of the validated 7 lncRNAs panel as diagnostic biomarkers in UCEC, providing new insights into the UCEC pathogenesis.

## Introduction

Uterine Corpus Endometrial Carcinoma (UCEC), also referred to as endometrial cancer, is one of the three major malignant tumors of the female reproductive system. It is the second most prevalent malignant tumor (20~30%) of the female reproductive system in China 1. Among the malignant tumors of the female reproductive system, UCEC has a better prognosis with a 5-year survival rate of 74% to 91% 2, 3. However, it is important to further improve the survival rate with the development of reliable and accurate tumor markers for UCEC for early diagnosis. The current treatment of UCEC is mainly surgery, supported by radiotherapy, chemotherapy, and targeted therapy 4, 5. The gold standard for diagnosis is segmental curettage and hysteroscopic endometrial biopsy. However, there are many parameters that may influence the results. For example, the size or location of the tissue sampling may not meet the detection standards. As a result, a single test may not provide a clear diagnosis and it is difficult to achieve the purpose of dynamic monitoring of the disease through multiple sampling 6. Serum tumor markers were commonly used in clinical diagnosis of UCEC include CA125, CA199, HE4, however their specificity and sensitivity for detecting early-stage tumors remain relatively low 7. Therefore, effective biomarkers to provide the basis for early diagnosis, treatment, and prognosis of UCEC tumors are urgently needed.

In the past few years, there has been a surge in interest in studying long non-coding RNAs. One popular approach to visualize the complex transcriptional regulatory interactions between microRNAs (miRNAs), messenger RNAs (mRNAs), and lncRNAs is by constructing a competitive endogenous RNA (ceRNA) network 8, 9. The ceRNA hypothesis, also known as the miRNA sponge, was proposed by Salmena et al. in 2011, which elucidates the intrinsic mechanism by which various RNAs regulate biological processes across transcriptomes 10, 11. LncRNAs are endogenous RNAs with more than 200 nucleotides without open reading frames and the ability to encode proteins. They provide the binding sites for protein or interact with DNA and RNA through complementary base pairing to regulate multiple biological processes 12. It has been known that lncRNAs participate in chromatin remodeling, post-translational modifications 13, histone and splicing modifications and other processes in the form of RNAs 14, 15. They are also able to form nucleic acid-protein complexes or become small RNA molecules to exert different biological functions 16. Studies have shown that lncRNAs can regulate the expression of oncogenes and tumor suppressor genes through epigenetic transcriptional regulation, post-transcriptional regulation and other mechanisms, leading to gene activation or silencing 17, 18. It can also act as a regulation of ceRNA and miRNA to mediate the expression level of target genes through the competitive binding of miRNA response elements (MREs) which play an important role in tumorigenesis 19.

The relationship between lncRNAs and UCEC has been suggested in different aspects including the mechanisms by which lncRNA functions. For example, oncogenic lncRNAs promote the occurrence and development of tumors by enhancing cell proliferation and invasion and inhibiting apoptosis 20. On the other hand, tumor suppressor lncRNAs inhibit tumor cells' proliferation, migration and invasion, and metastasis. They also promote cell apoptosis and inhibit tumorigenesis and development 21. Studies have suggested that lncRNAs are potential diagnostic biomarkers for UCEC. For instance, Ouyang et al. constructed a model based on seven lncRNAs (AC110491.1, AL451137.1, AC005381.1, AC103563.2, AC007422.2, AC108025.2, and MIR7-3HG) as potential prognostic factors. The patients were categorized into high- and low-risk groups by this model, and survival was significantly improved in the low-risk group 22. Moreover, lncRNAs can be also used to assess the prognosis of UCEC. For example, the expression level of lncRNA UCA1 in lymph node metastasis tissues is higher than that in proliferative endometrium and primary UCEC tissues. The expression level of UCA1 is closely related to lymph node metastasis, distant metastasis, tumor grade, advanced TNM and vascular invasion and has become a reference indicator for determining the prognosis of patients with endometrial cancer 23. Furthermore, lncRNAs can predict the response to chemotherapy. Sun found that the expression level of lncRNA HOTAIR in cisplatin-resistant UCEC cells was significantly reduced 24. Therefore, lncRNAs can be used as molecular markers for predicting the efficacy of treatments and drug resistance in UCEC.

Given there is a lack of comprehensive studies on lncRNAs in UCEC, we used lncRNA, mRNA and miRNA expression data from The Cancer Genome Atlas (TCGA) to construct a ceRNA network. Clinical samples were also retrieved from our hospital for validating the bioinformatics results. The mechanism of action of lncRNAs and their clinical value in early diagnosis and treatment of UCEC is investigated in this study to provide a new insight into the mechanism of UCEC pathogenesis. We mainly focused on lncRNAs of the ceRNAs involved in cancer processes. Finally, a set of lncRNAs (C20orf56, LOC100144604, LOC100190940, LOC151534, LOC727677, FLJ35390, LOC158572) was found to be a potential prognostic indicator in UCEC.

## Materials and Methods

### Data mining and samples collection

**Figure 1:**
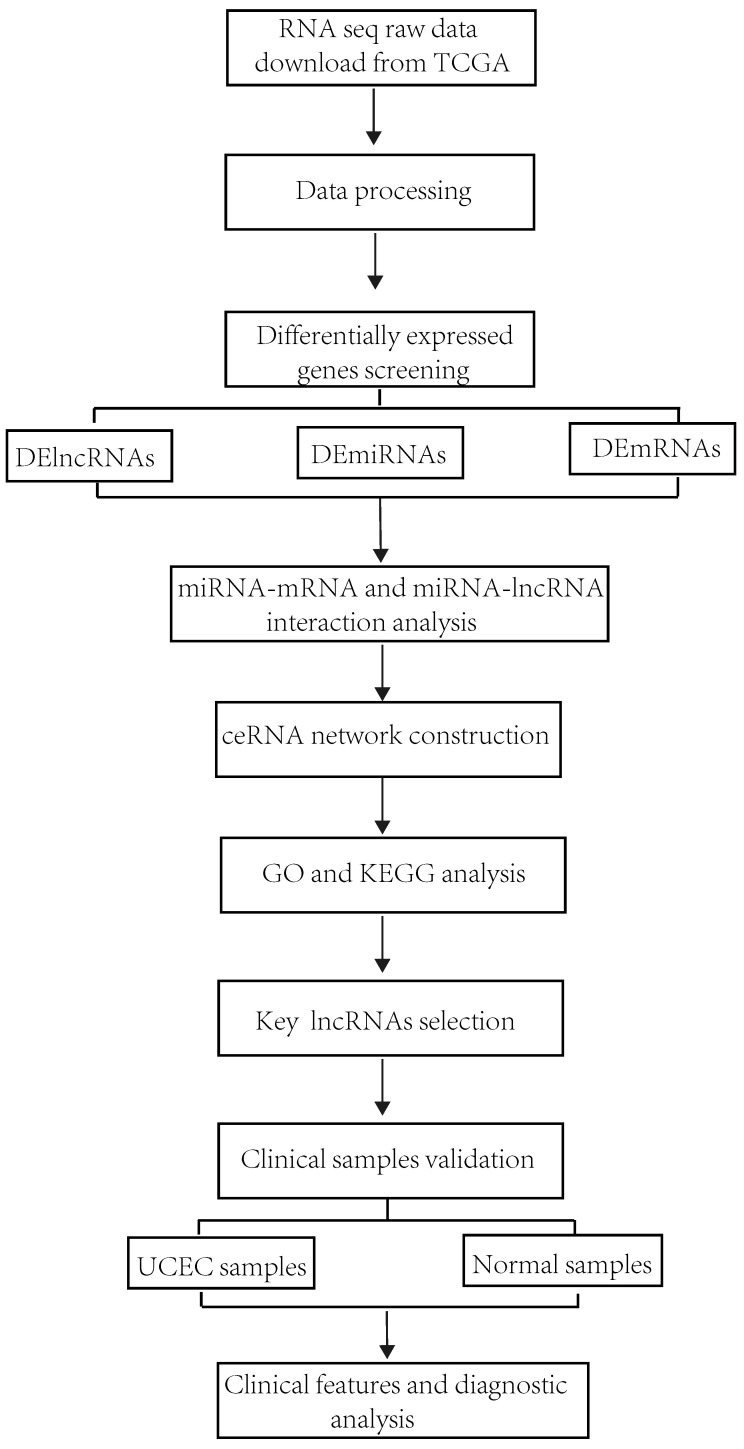
Flowchart of the construction of ceRNA network in UCEC.

This study's workflow is shown in Figure 1. Clinical information and RNA-sequencing (RNA-seq) data were downloaded from The Cancer Genome Atlas (TCGA) database (https://portal.gdc.cancer.gov/)) by RTCGA Toolbox R package 25. The RNA-seq dataset is composed of three types of data including lncRNAs, mRNAs and miRNA. According to the exclusion criteria, the samples were excluded if 1) their histological diagnosis was not UCEC; 2) they contained other malignant tumors; 3) incomplete clinical data were provided; 4) they were obtained from patients treated with radiotherapy and chemotherapy before surgery.

The tissues of patients with endometrial cancer were diagnosed by the Department of Gynecology of Yunnan Cancer Hospital. The study design was approved by the appropriate ethics review board of Committee of Yunnan Cancer Hospital (No. KYLX202146). All patients were informed consent. A total of 38 patients were collected as the case objects, and 10 relatively normal endometrium of non-UCEC patients were collected as the control group. All cases were from the first onset, without any treatment before surgery, and no history of other malignant tumors. The specimens were confirmed by pathological diagnosis. Intraoperative endometrial tissue specimens were placed in a cryotube containing GeneFresh Tissue ATCG RT Storage solution (GeneDoTech, Shenzhen). All specimens were transferred to the refrigerator at -70°C for storage. Basic information and clinical data of patients with endometrial cancer were collected, including age, menopause status, tumor's location, tumor's size, pathological type, FIGO stage, degree of differentiation, lymph node metastasis and distant metastasis, and follow-up treatment methods after surgery.

### Differential expressed genes screening and analysis

The RNASeqV2 system was used to process the raw data of lncRNA and mRNA 26. The raw data of microRNA sequencing was normalized by Illumina HiSeq 2000 miRNA seq sequencing platforms' high throughput sequencing software. R Studio was used to analyze the difference in expression levels between tumor tissues and normal controls and calculate the False Discovery Rate (FDR), absolute log2 Fold Change (log2FC) and P values. Differentially expressed (DE) miRNAs, lncRNAs and mRNAs were obtained by the thresholds of absolute log2FC > 1.5, FDR < 0.05, and P value < 0.01. We plotted heatmaps and volcano plots using the R package "ggplots" to visualize the results of the differential expression analysis 27.

### Construction of ceRNA network of UCEC

To better explore the relationship between DE lncRNAs, miRNAs and mRNAs, the TCGA database was used to construct a lncRNA-mediated ceRNA network in UCECs. First, miRcode (http://www.mircode.org/) was used to predict the miRNAs that interact with differential expressed lncRNAs, and intersect them with the DE miRNAs 28. Then, the three databases, Targetscan (http://www.targetscan.org/) 29, miRDB (http://www.mirdb.org/) 30 and miRTarBase (http://mirtarbase.mbc.nctu.edu.tw/) 31 were used for comparative analysis to identify the relationship between the DE miRNAs and the DE mRNAs, while the mRNAs targeted by miRNAs were predicted. After a series of analyses, the lncRNA-miRNA and miRNA-mRNA regulatory pairs were identified. Finally, using Cytoscape (http://www.cytoscape.org/), the corresponding relationships between the three were visualized to construct a ceRNA network diagram 11. Additionally, mRNAs of tumor suppressor genes (TSGs) and oncogenes were retrieved from the Network of Cancer (NCG) database (v7.1), including 254 TSGs and 256 oncogenes. mRNAs of apoptosis-related genes were retrieved from the Reactome database with the pathway ID “R-HSA-109581) which contains 181 genes for analysis.

### Gene Ontology and the Kyoto Encyclopedia of Genes and Genomes pathway enrichment analysis

The Database for Annotation Visualization and Integrated Discovery (DAVID) (https://david.ncifcrf.gov/) was used to perform functional and pathway enrichment analyses for the lncRNA-related DEmRNAs in the ceRNA network 32. “Human” was selected in the Gene Ontology (GO) database and the GO terms with p-value <0.05 and enrichment scores > 1.5 were considered as significantly enriched. Using the same screening criteria and methods, the significantly enriched pathways of DE genes were identified using the Kyoto Encyclopedia of Genes and Genomes (KEGG) database.

### Survival analysis based on differential expressed lncRNAs

The Kaplan‐Meier survival analysis was used to evaluate the association between differentially expressed lncRNAs (C20orf56, LOC100144604, LOC100190940, LOC151534, LOC727677, FLJ35390, LOC158572) and overall survival of the patients 33. Survival curve was generated using the R package "survival".

### RNA extraction and qRT-PCR validation

Total RNA was extracted from tissues using the RNA extraction kit from Shenzhen Genedo Medical Technology Co., Ltd. (Shenzhen, China), which was reverse transcribed into cDNA using a reverse transcription kit (Takara, Dalian, China). Next, qRT-PCR was performed using the FastStart Universal SYBR Green Mastermix (Takara, Dalian, China) on LightCycler® 480 instrument. All the PCR results were calculated using the -ΔCt method, where -ΔCt =- (Ct _lncRNA_ - Ct _GAPDH_) tumor, and 2-ΔCt represents fold change. The qRT-PCR reactions were repeated in triplicate. Primers for qRT-PCR were synthesized by Shanghai Integrated Biotech Solutions Co.,Ltd (Shanghai, China), and the primers sequences could be found in Table 1.

### Construction of prognostic model by validated lncRNAs

To assess the performance of lncRNAs, we used the R package pROC to plot and visualize receiver operating characteristic (ROC) curves and compute the area under the curve (AUC) and its confidence intervals 34. LASSO (least absolute shrinkage and selection operator) regression model was fitted to the lncRNA-based classifier using the R package glmnet via penalized maximum likelihood 35.

### Clinical features analysis of key lncRNAs

We selected the lncRNAs from the subnetwork to study their associations with specific clinical characteristics of the patients, including age, menopause status, tumor's location, tumor's size, pathological type, FIGO stage, degree of differentiation, lymph node metastasis and distant metastasis, and tumor biomarker value. The patients were divided into two groups according to the clinical features cut-off value. The lncRNA expression levels were analyzed for statistical significance of the difference using Student's t test for independent samples by two-group comparisons.

### Statistical analysis

R Studio (R version 3.4.1), GraphPad Prism 8.2 and SPSS 19.0 statistical packages were used for statistical analysis. The log-rank test was used in the Kaplan-Meier survival curve analysis, and the Student's t test (two-tailed) was used in qRT-PCR analysis between two groups of data sets. Results with P-value <0.05 were considered statistically significant.

## Results

### Screening of differentially expressed RNA genes in UCEC

**Figure 2:**
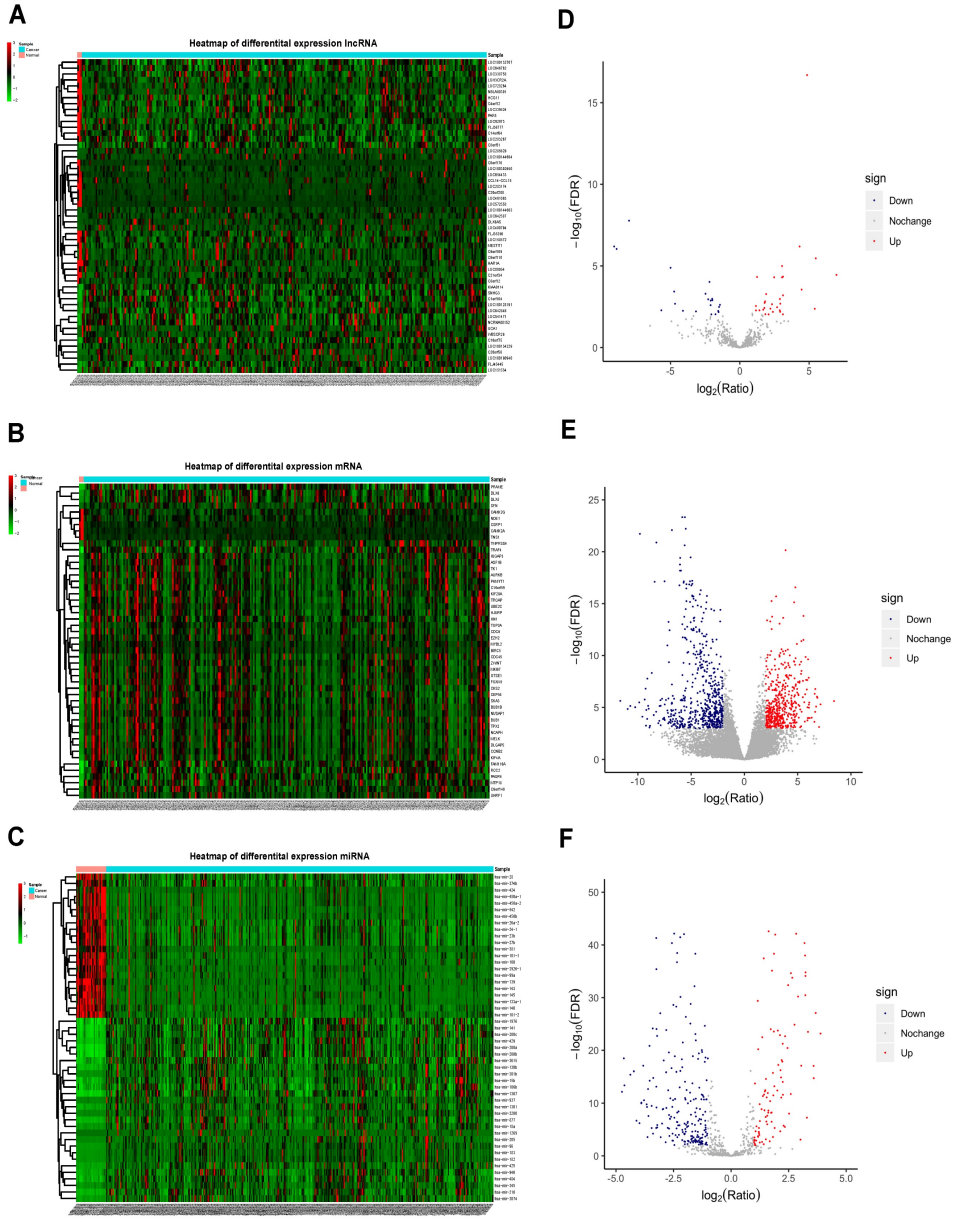
** Heatmap and volcano plots for differentially expressed mRNAs, miRNA and lncRNAs.** Left panels, heat maps for all differentially expressed lncRNAs (A), mRNAs (B), and miRNAs (C) in UCEC; Right panels, volcano plots showing lncRNAs (D), mRNAs (E), and miRNAs (F) with |log2FC| ≥ 1.5 (P < .001). Blue, downregulated; red, upregulated; gray, not differentially expressed. lncRNA: long noncoding RNA; miRNA: microRNA

The normalized data obtained from TCGA contained protein-coding RNAs, non-coding RNAs, pseudogenes, immunoglobulins, and other non-coding RNAs. Relevant UCEC data were retrieved from the TCGA database, with a total of 266 UCEC samples and 3 control samples which met the eligibility criteria included for analysis. In total, 53 differentially expressed lncRNAs (DElncRNAs) (31 upregulated and 22 downregulated), 1072 differentially expressed mRNAs (DEmRNAs) (477 up-regulated and 595 down-regulated), and 318 differentially expressed miRNAs (DEmiRNAs) (100 upregulated and 218 downregulated) were identified. The top 50 DElncRNAs, DEmRNAs and DEmiRNAs are shown in Figure 2A, 2B and 2C and the volcano plots are shown in Figure 2D, 2E and 2F. The top 10 mRNAs, lncRNAs, and miRNAs exhibiting significant upregulation and downregulation are listed in Table 1.

### Construction of the miRNA-lncRNA-mRNA ceRNA network in UCEC

**Figure 3:**
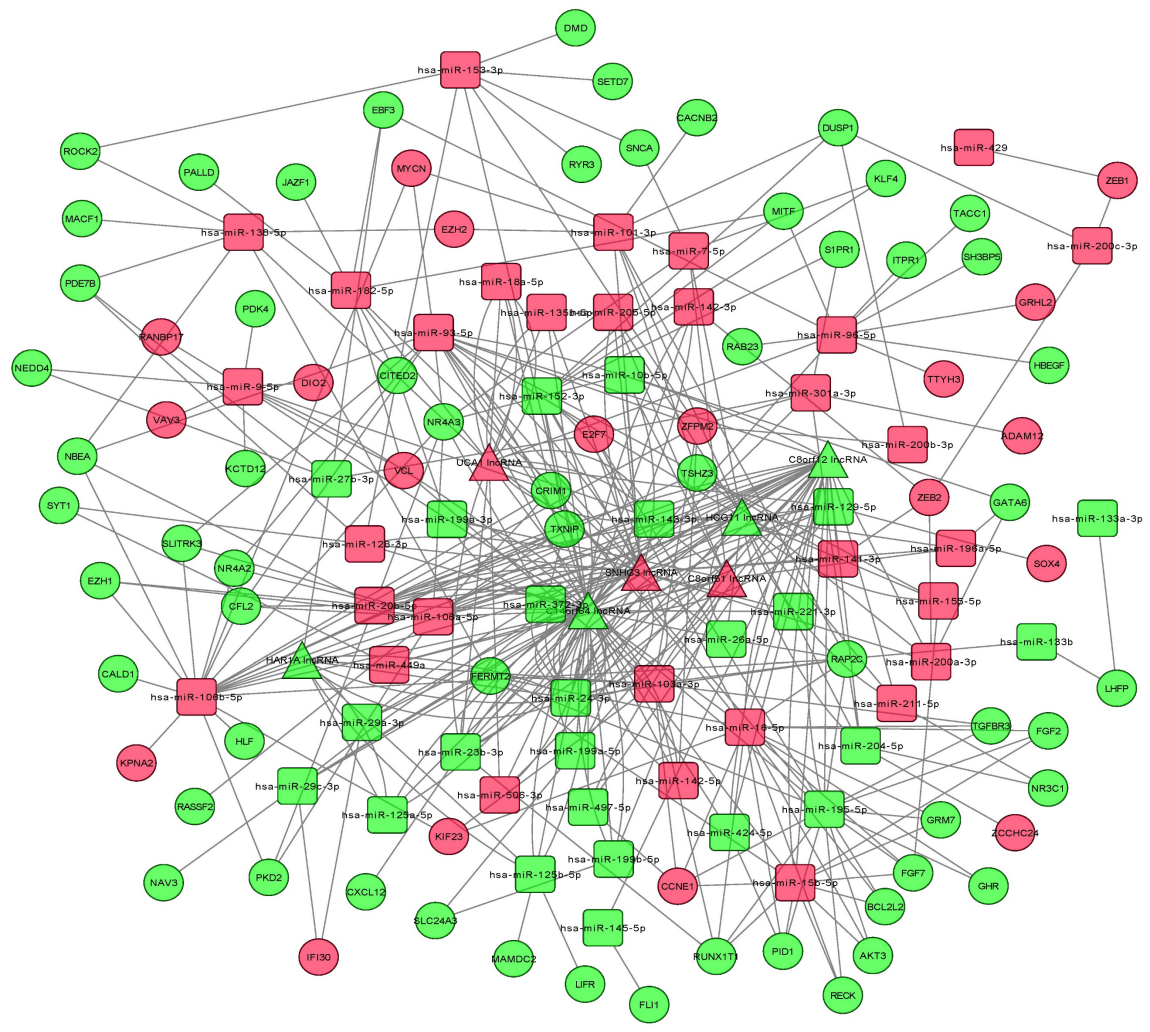
** The lncRNA-miRNA-mRNA competitive endogenous (ceRNA) network.** Squares represent miRNA, circles represent mRNAs, triangles represent lncRNAs.

StarBase v2.0 was used to explore potential microRNA response elements (MREs) and predict the relationship between lncRNAs and miRNAs. The miRCode database was used to predict the target lncRNAs of the DEmiRNAs, and the list of relationships between them was obtained. The analyses identified a total of 503 lncRNA-miRNA regulatory pairs consisting of interactions between 97 DEmiRNAs and 15 DElncRNAs while 733 miRNA-mRNA regulatory pairs were composed of interactions between 97 DEmiRNAs and 250 DEmRNAs (Supplementary Tables S1 and S2). Additionally, the relationships between the DEmRNAs of oncogenes, tumor suppressor genes (TSGs) and apoptosis-related genes and DEmiRNAs, DElncRNAs were studied (Supplementary Table S3). A total of 8 mRNAs of TSGs were found to be interacting with 13 DEmiRNAs and 13 DElncRNAs, 16 mRNAs of oncogenes were associated with 40 DEmiRNAs and 14 DElncRNAs, while 5 mRNAs of apoptosis-related genes were related to 9 DEmiRNAs and 11 DElncRNAs. In order to further understand the functions of DElncRNAs, the regulatory ceRNA network of the competitive target relationship between the DElncRNAs, DEmRNAs and DEmiRNAs was constructed that includes 7 lncRNAs, 105 mRNAs and 94 miRNAs (Figure 3).

### Functional enrichment analysis the ceRNA network-associated DEmRNAs

**Figure 4:**
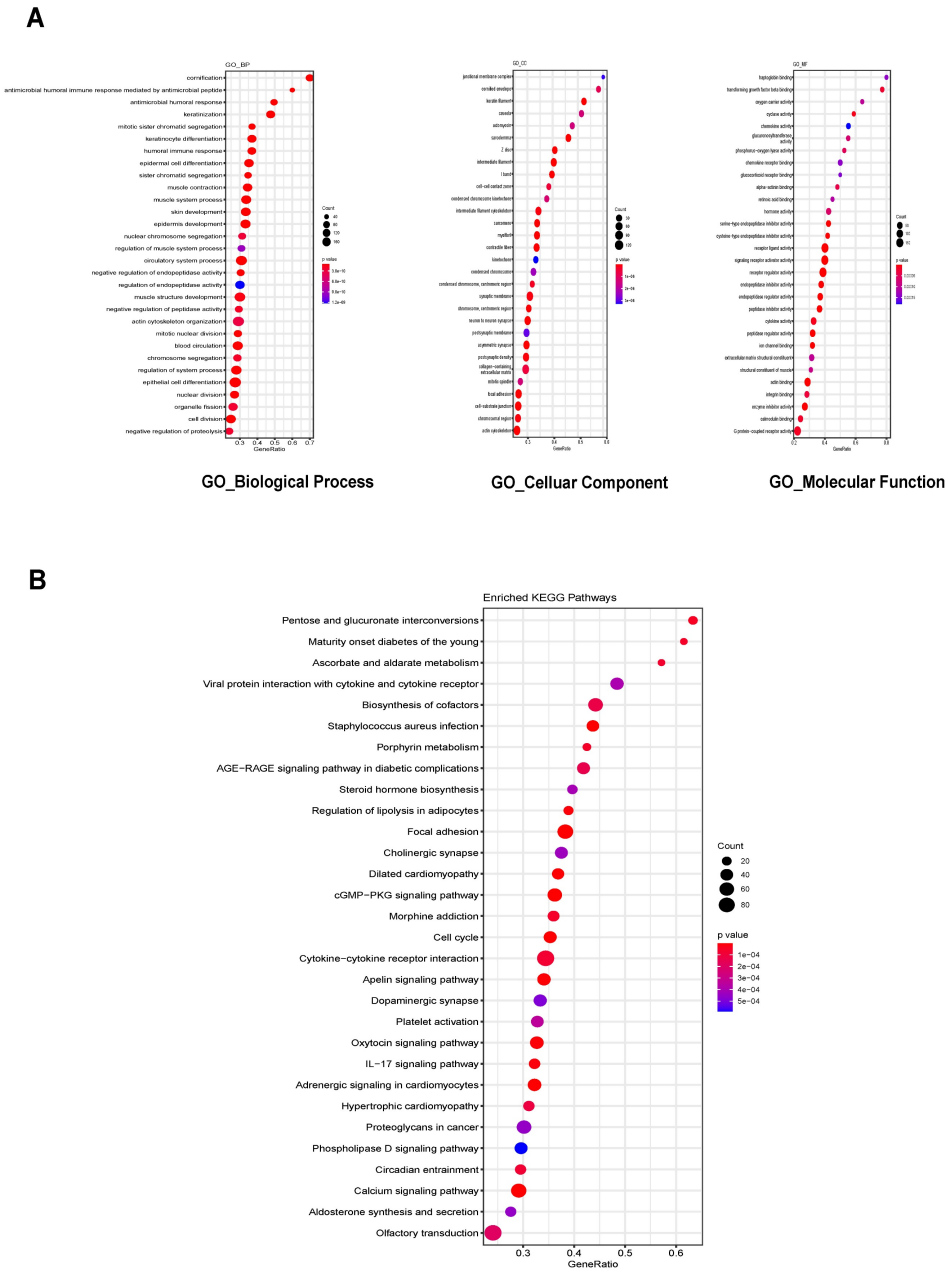
** The enrichment analysis of GO and KEGG pathway. (**A) Top 30 GO biological process terms, Cellular Components terms and Molecular function terms of the DEmRNAs in the ceRNA network (B) Top 30 KEGG pathways of DEmRNAs in the ceRNA network. GO: Gene Ontology; KEGG: Kyoto Encyclopedia of Genes and Genomes.

We then predicted the roles of lncRNAs by analyzing ceRNA network-associated DEmRNAs. Gene Ontology (GO) analysis was performed separately for Biological Process (BP), Cellular Component (CC) and Molecular Function (MF). The top 30 enriched GO terms based on the p-value were identified, including the regulation of epidermal cell differentiation for BP, focal adhesion for CC, and signaling receptor activator activity for MF (Figure 4A). Kyoto Encyclopedia of Genes and Genomes (KEGG) enrichment analysis was also performed, with the top 30 enriched pathways identified including biosynthesis of cofactors, cytokine-cytokine receptor interaction and cGMP-PKG signaling pathways (Figure 4B). Our findings provide a valuable resource for discovering additional molecular participants/interactions in UCEC, as these functions have not been studied in UCEC to our knowledge.

### Kaplan-Meier survival analysis of DElncRNAs

**Figure 5:**
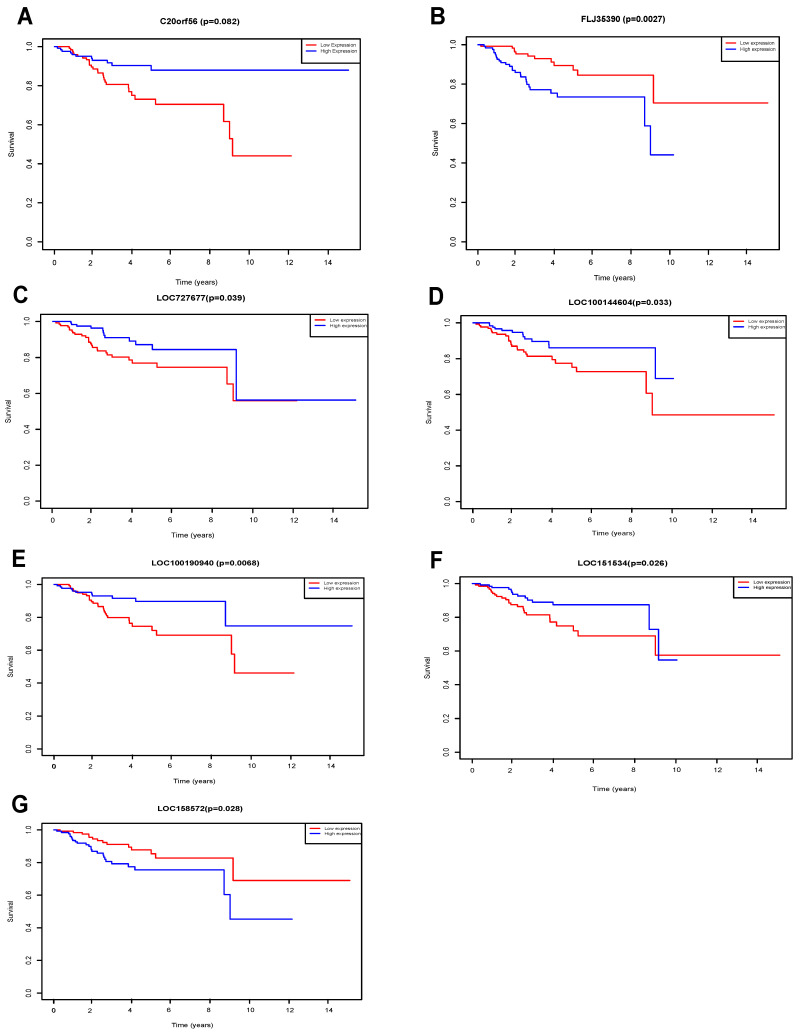
** KM survival of DElncRNAs.** Survival curves for DElncRNAs that are associated with the overall survival (OS) of UCECpatients. (A) C20orf56, (B) FLJ35390, (C) LOC727677, (D) LOC100144604, (E) LOC100190940, (F) LOC151534, (G) LOC158572.

Seven lncRNAs (C20orf56, LOC100144604, LOC100190940, LOC151534, LOC727677, FLJ35390, LOC158572) were identified as being significantly associated with the overall survival of UCEC patients by Kaplan-Meier survival analysis and log-rank test (p<0.05) (Figure 5). We found that high expression of the 4 lncRNAs (FLJ35390, LOC100144604, LOC151534 LOC158572) was associated with poorer prognosis in UCEC patients. In contrast, high expression of 3 lncRNAs (C20orf56, LOC100190940, LOC727677) was associated with better prognosis in UCEC patients.

### Validation of DElncRNAs in clinical samples

**Figure 6:**
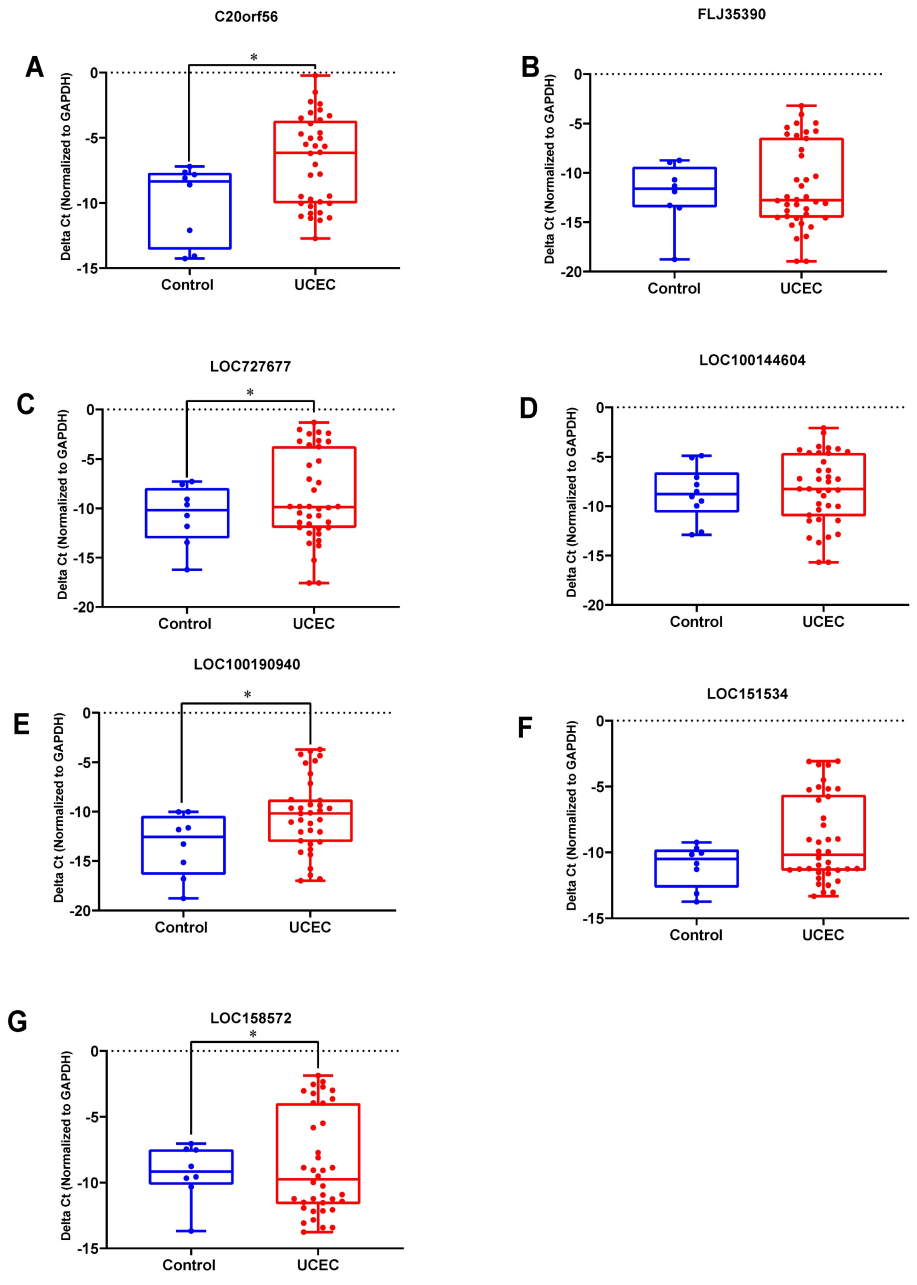
** The expression level of 7 lncRNAs in UCEC and normal tissues.** DElncRNAs were detected using qRT-PCR. (A) C20orf56, (B) FLJ35390, (C) LOC727677, (D) LOC100144604, (E) LOC100190940, (F) LOC151534, (G) LOC158572.UCEC represents UCEC patient group, Control represents the normal tissue group. Experiments were performed in triplicate. * p<0.05 by Student's t test.

qRT-PCR experiments were used to further validate the expression levels of the 7 key lncRNA genes in 38 UCEC samples and 10 normal endometrial tissues. The expression levels of these lncRNAs are shown in Figure 6. Four lncRNAs (C20orf56, LOC727677, LOC100190940 and LOC158572) were significantly up-regulated in the UCEC samples compared to the control group (Figure 6A, 6C, 6E, 6G). However, there was no significant difference for the three lncRNAs (FLJ35390, LOC100144604 and LOC151534) between the two groups (Figure 6B, 6D, 6F).

### Correlation analysis of lncRNA expression levels in UCEC patient tissues and clinical data

**Figure 7:**
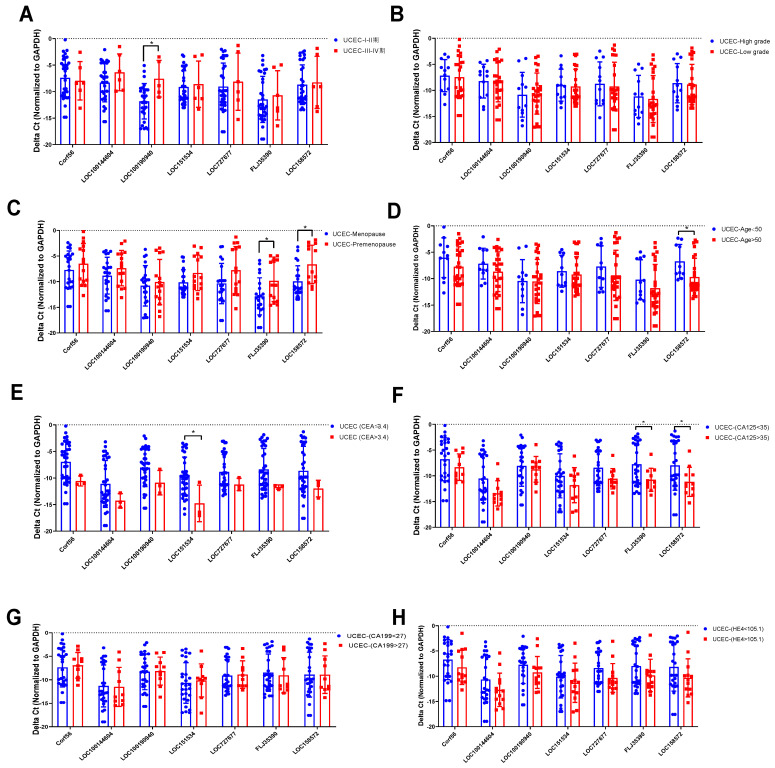
** The analysis of 7 lncRNAs expression level and clinical features.** DElncRNAs expression levels were compared between the two groups divided according to clinical features cut-off value (A) Tumor stage, (B) Tumor grade, (C) Menopause status, (D) Age, (E) CEA, (F) CA125, (G) CA199, (H) HE4. Experiments were performed in triplicate. * p<0.05 by Student's t test.

The relationship between the expression levels of 7 lncRNAs in 38 UCEC samples and the clinicopathological characteristics (age, menopause, stage, differentiation, etc.) of endometrial cancer patients was analyzed. The overall correlation between lncRNA expression level and clinical features were listed in Table 3. qRT-PCR was used to assess the correlation between lncRNA expression and clinical features. In clinical staging, the expression of LOC100190940 in endometrial tissue of patients with stage III-IV UCEC was higher than that of patients with stage I-II (Figure 7A). There was no significant difference in the degree of differentiation of different pathological tissues (Figure 7B). The expression levels of FLJ35390 (P=0.0268) and LOC15857 (P=0.0080) in the premenopausal group were higher than those in the postmenopausal group (Figure 7C), while the expression level of LOC158572 in the young (aged ≤50) group was higher than that in the old (aged >50) group (Figure 7D). Moreover, the expression level of LOC151534 was significantly lower in high CEA (>3.4) group than that in low CEA (<3.4) group (Figure 7E), while the expression levels of FLJ35390 and LOC58572 were significantly lower in high CA125(>35) group than that in low CA125 (<35) group (Figure 7F). Among the tumor markers currently used to for the diagnosis of UCEC, the expression of 7 lncRNAs was not significantly associated with the serum levels of CA199 and HE4 (Figure 7G, 7H).

### Construction of prognostic model by seven key lncRNAs

**Figure 8:**
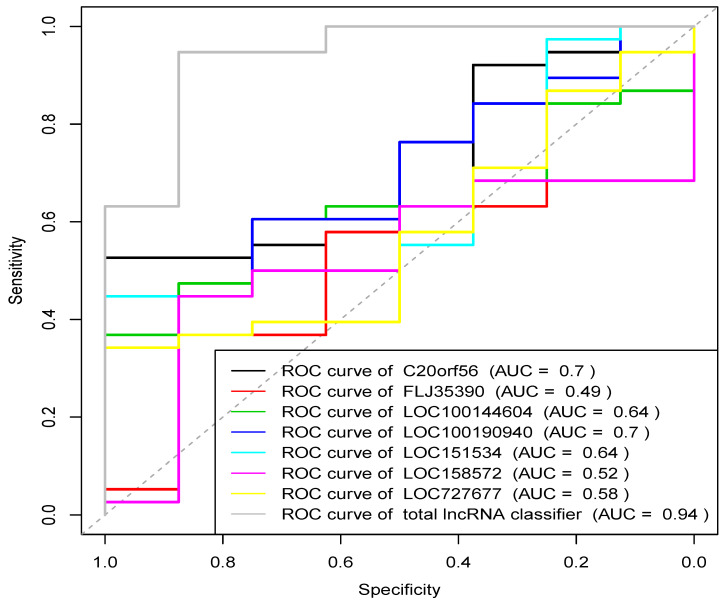
Receiver operating characteristic (ROC) curve of 7 lncRNAs.

Based on the above experimental results, lncRNAs with statistically significant differences in expression between UCEC and control groups (P<0.05) were extracted to analyze their diagnostic value for UCEC. Receiver operating curves (ROC) were drawn based on the expression data of the seven identified lncRNAs, as shown in Figure 8. The ROC analysis showed that the results were statistically significant (P<0.05). Except for FLJ35390, the other six lncRNAs were able to distinguish UCEC and normal endometrial patients. Logistic regression modeling was used to fit the statistical results of the 7 lncRNAs into a combined detection data, and the ROC curve analysis was used to obtain the combined detection results. The area under the curve (AUC) was 0.941 (95% CI: 0.875-0.947). Our result suggests that the combined detection of 7 lncRNAs has a relatively greater diagnostic value for distinguishing endometrial cancer samples from normal endometrial tissues.

## Discussion

Endometrial cancer is the second most common malignant tumor of female reproductive system in China. Although many advances have been made in biomedical research on the molecular mechanisms and treatments of UCEC, the overall survival rate of patients remains low since most of the patients are in the advanced stage at the time of diagnosis with poor surgical outcomes and prognosis 36. UCEC is the leading gynecologic malignancy in developed nations, with about 7% of cases occurring in women under 45. Standard treatments, which involve hysterectomy and salpingo-oophorectomy, often clash with the fertility desires of younger patients. Therefore, a fertility-sparing approach, suitable for early-stage and low-grade endometrial cancer patients, is essential. The efficacy of this approach is studied through the evaluation of various immunohistochemical markers and their response to hormonal therapy 37, 38.

Circulating miRNAs were reported as a promising avenue for early EC diagnosis, staging, and evaluating a woman's receptivity, providing a non-invasive method with reduced error margins. This miR-based approach could be a pivotal tool in fertility-preserving processes. However, the ethical, legal, and regulatory considerations of such innovations need to be addressed alongside their potential benefits 39. Oocyte vitrification is also a method of fertility preservation for couples diagnosed with UCEC. Upon diagnosis, they undergo ovarian stimulation to retrieve mature oocytes, which are then frozen for future use. Oocytes that have undergone vitrification appear to possess comparable fertilization and implantation potential as fresh oocytes. Collaboration between oncologists and fertility experts ensures optimal cancer treatment while safeguarding reproductive options 40, 41. Currently, the serum tumor markers used for diagnosis of UCEC include CEA, CA125, CA199, and HE4 42. However, the diagnostic efficiency of these methods is hampered by their limited sensitivity and specificity. Therefore, there is an urgent need to identify novel biomarkers of UCEC that are non-invasive, specific, and sensitive for early diagnosis and treatment of the disease. In our study, this study identified specific lncRNAs by constructing the ceRNA network of UCEC using data obtained from the TCGA database, compared and validated the results from bioinformatics analysis using clinical tissue samples. First, 266 UCEC samples and 3 normal control samples were retrieved from the TCGA database and analyzed by RNA sequencing to obtain the raw data of miRNA, mRNA and lncRNA expression. Then, a ceRNA regulatory network was constructed to predict the relationships between them to screen for potential UCEC-related lncRNA biomarkers. Moreover, several interactions between mRNAs, miRNAs and lncRNAs were observed that involve the mRNAs of TSGs, oncogenes, and apoptosis-related genes. These interactions provide an additional direction that they may contribute to tumorigenesis and tumor cell survival which is worth further investigation in future studies. The 250 differentially expressed genes (DEmRNAs) identified were then subjected to GO and KEGG functional enrichment analysis and annotation, and the top 30 enriched biological processes and signalling pathways were selected. The results showed that the most enriched GO terms of DEmRNA include the processes of the muscle system, the cell-cell junction and actin binding. The KEGG enrichment analysis revealed predominantly cancer-related pathways such as cGMP-PKG signaling pathways. In order to validate the potential lncRNA biomarkers of UCEC screened based on the ceRNA network, we selected seven lncRNAs (C20orf56, LOC100190940, LOC100144604, LOC727677, LOC151534, LOC158572, FLJ35390) with significantly different expression in the ceRNA gene network.

We used qRT-PCR to detect the expression levels of 7 lncRNAs genes in the UCEC samples and normal endometrial tissue, and correlated them with the clinicopathological characteristics of patients with endometrial cancer. Our results showed that these lncRNAs are involved in the differentiation and proliferation of endometrial cancer tumor cells and may play an important role in the malignant transformation process of endometrial cancer, which may aid in the early diagnosis of endometrial cancer. However, further research is needed to investigate the mechanism of expression regulation in the cancer. The results of ROC analysis also showed that the combined detection of the 7 lncRNAs has greater diagnostic value in distinguishing endometrial cancer samples from normal endometrial tissues. The up-regulated expression of the five lncRNAs was consistent with the results of the bioinformatics analysis. It was found that the expression level of LOC100190940 was higher in stage III and IV UCEC patients than in stage I and II patients. Moreover, the expression level of LOC158572 was negatively correlated with patient's age. The expression levels of FLJ35390 and LOC15857 in premenopausal patients were higher than those in postmenopausal patients. As a well-studied lncRNA, C20orf56 (LINC00261) has been widely studied in pancreatic cancer, prostate cancer, non-small cell lung cancer, bile duct cancer, colon cancer, endometrial cancer and other tumors. It may be used as early diagnosis, prognosis and target indicators of treatment. The results of this study also showed that LINC00261 was significantly up-regulated in UCEC samples which is in line with the literature's findings. LINC00261 can also be over-expressed in pancreatic cancer tissues by binding miR-222-3p to activate the HIPK2/ERK/c-myc pathway 43, which unmasked a new epigenetic and post-transcriptional regulatory mechanism that contributes to targeted therapy for pancreatic cancer. Another study showed that the down-regulation of LINC00261 expression was considered to be an independent risk factor affecting the postoperative recurrence-free survival rate of colon cancer patients (P<0.05) 44. Being significantly correlated with clinical stage, LINC00261 may serve as a novel molecular biomarker for predicting colon cancer metastasis and survival. The expression level of LOC100190940 is positively correlated to the clinical stage of endometrial cancer and its high expression is closely related to the metastasis of UCEC. As a result, it may be used as an early diagnosis indicator of endometrial cancer and provides a direction for future research. Currently, LOC100190940 has not been reported in UCEC while other literatures have found that LOC100190940 can promote the occurrence and development of colorectal cancer and lung cancer. In colorectal cancer, LINC02418 negatively regulates apoptosis through the LINC02418/miR-34b-5p/BCL2 axis, acts as a tumor driver and can be used as an indicator for predicting prognosis 45; The expression levels of LOC151534 (LBX2-AS1) and FLJ35390 (LINC00957) were down-regulated in UCEC based on the data obtained from TCGA, and their expression levels were higher in premenopausal patients than in postmenopausal patients, which also suggests their effects as tumor suppressor. In this study, the expression level of LOC151534 (LBX2-AS1) was also significantly correlated with the age of patients with UCEC. However, due to the small sample size of the experiment, the difference was not obvious and further confirmation was needed through increasing the sample size. In this study, it was found that LncRNA LBX2-AS1 was identified as an oncogene in some tumors. It is also abnormally expressed in liver cancer 46, non-small cell lung cancer 47 and associated with tumor cell proliferation, migration and invasion. It may also act as a novel prognostic biomarker and therapeutic target. Tumor biomarkers have extremely important clinical application value in disease screening, monitoring recurrence and metastasis as well as evaluating treatment outcome and prognosis of patients.

## Conclusion

Based on our results, 7 potential lncRNA biomarkers (C20orf56, LOC100144604, LOC100190940, LOC151534, LOC727677, FLJ35390, LOC158572) were validated in UCEC patients by quantitative real-time PCR. We further analyzed the correlation between these lncRNAs and tumor biomarkers and found that the expression levels of FLJ35390 and LOC15857 in UCEC were correlated with that of CA125 while LOC151534 was correlated with CEA. Therefore, they can reflect the onset, differentiation, and disease progression of UCEC and are of great value in diagnosis, treatment outcome monitoring and prognosis evaluation of UCEC. However, the number of clinical samples collected in this experiment is small and the results need to be further validated by increasing the number of samples to improve the reliability of the results. In this study, LOC100190940 and LOC158572 are considered as novel RNA molecules with unknown functions reported. Future studies will employ larger sample size for confirming our findings.

## Supplementary Material

Supplementary tables.

## Figures and Tables

**Table 1:** Top 10 upregulated and downregulated miRNAs, lncRNAs, and mRNAs in UCEC

Differentially expressed lncRNAs				Differentially expressed mRNAs				Differentially expressed miRNAs		
Gene symbol	log_2_FC	P value		Gene symbol	log_2_FC	P value		Gene symbol	log_2_FC	P value
Upregulation				Upregulation				Upregulation		
LOC642587	9.067395	1.35E-08		DEFA5	23.45222	2.99E-07		hsa-mir-1269	8.30602	1.82E-42
C20orf56	8.891294	2.03E-08		MAGEA9B	22.23204	2.48E-16		hsa-mir-205	5.937647	1.91E-63
UCA1	7.99487	2.22E-10		RPTN	21.45037	2.65E-11		hsa-mir-516a-2	5.424192	1.83E-17
DSCR8	6.476508	0.011553		POU3F3	21.31608	4.67E-10		hsa-mir-183	4.898356	1E-127
LOC400794	5.662909	0.0007		AMBN	20.87678	1.42E-10		hsa-mir-522	4.739051	1.21E-13
DLX6AS	4.997421	4.6E-07		MT4	20.8689	6.97E-08		hsa-mir-519a-1	4.658677	3.55E-20
C12orf36	4.934214	0.005073		MAGEA3	20.83983	8.38E-10		hsa-mir-516a-1	4.632546	6.43E-15
LOC285629	4.738387	2.61E-05		SPINK6	20.80407	1.18E-11		hsa-mir-138-2	4.375384	5.4E-17
LOC100190940	4.674075	0.000215		CST1	11.65061	6.15E-08		hsa-mir-96	4.318775	5.9E-107
MGC4473	4.474938	0.010739		CST4	10.95447	5.17E-07		hsa-mir-891a	4.224868	1.21E-17
Downregulation				Downregulation				Downregulation		
LOC572558	-6.97616	1.5E-06		SLITRK3	-8.4095	6.44E-08		hsa-mir-1-2	-3.88584	5.31E-25
LOC283174	-5.47984	9.36E-08		BCHE	-7.14543	1.54E-07		hsa-mir-133a-1	-3.67418	5.06E-29
C6orf176	-5.40818	0.000527		TCF23	-7.00746	6.19E-05		hsa-mir-133a-2	-3.59272	2.53E-16
LOC401093	-4.85392	2.96E-20		C7	-6.85244	2.75E-08		hsa-mir-133b	-3.5841	1.12E-18
CCL14-CCL15	-4.45833	1.87E-05		PTGER3	-6.81345	2.06E-08		hsa-mir-1247	-3.34065	2.51E-25
C20orf200	-4.31234	1.36E-08		PTGFR	-6.72977	2.72E-07		hsa-mir-1-1	-3.29388	1.58E-08
WIT1	-3.4896	0.004975		MYH11	-6.65421	3.04E-09		hsa-mir-424	-3.23424	6.98E-37
LOC255167	-3.19814	0.004812		SSTR3	-6.6446	1.1E-06		hsa-mir-100	-3.23122	3.58E-36
LOC100302650	-3.11601	2.04E-06		DPT	-6.59786	9.98E-09		hsa-mir-3926-1	-3.22531	1.83E-32
C9orf110	-3.11431	4.93E-05		DES	-6.57231	1.5E-05		hsa-mir-143	-3.2153	3.72E-40

**Table 2:** Real-time quantitative PCR primer sequences used in this study

Gene Symbol	Forward primer	Reverse primer	Product Length (bp)
GAPDH	ATCTCTGCCCCCTCTGCTGA	GATGACCTTGCCCACAGCCT	303
C20orf56	CCAAATGGGTGCTGTGTGTG	TACCATGGCAGCGTGATTGT	195
LOC100190940	ACTGTGGTCGCTGAGAACTG	GTTTCCGAGACCCACGTCAT	191
LOC100144604	ACCCCCAAGGAAGAGTCAGT	ACATGTCAGAAGCCGTCAGG	134
LOC727677	TATACACCAGAATGCCCCGC	CCATTGTCAACCGCAACACT	104
LOC151534	CGTGGGGAATGGACCCATAG	CGAGCCTTGGTCTTGTCTGT	118
LOC158572	TGAATCACGTGTGGAGGGTG	CCAGGTGCATCTACTGCGAA	173
FLJ35390	CAATACACGGGTGGGCAGAA	CTGGGCCCCATCATCAACAA	184

**Table 3:** Relationship between lncRNA expression levels and clinicopathological characteristics of UCEC patients

Clinicopathological characteristics	Sample size	Percentage	*P*-value						
			C20orf56	LOC100144604	LOC100190940	LOC151534	LOC727677	FLJ35390	LOC158572
**Degree of differentiation**									
**High-mid**	12	32.43%	0.8242	0.8861	0.8685	0.8244	0.7584	0.7569	0.8498
**Mid-Low**	25	67.57%							
**Clinical stage**									
**Stage I-II**	32	84.21%	0.5422	0.8252	0.0137	0.7135	0.6551	0.6948	0.8063
**Stage III-IV**	6	15.79%							
**Menopause status**									
**Menopause**	23	60.52%	0.3433	0.2207	0.5980	0.0725	0.0770	0.0268	0.0080
**Pre-menopause**	15	39.47%							
**Age**									
**≤50**	11	28.95%	0.2150	0.2325	0.9591	0.6004	0.3140	0.3019	0.0289
**>50**	27	71.05%							
**CEA**									
**<3.5**	35	92.11%	0.1174	0.3606	0.1892	0.0298	0.2341	0.1542	0.2246
**>3.5**	3	7.89%							
**CA125**									
**<35**	27	71.05%	0.2674	0.0615	0.6035	0.2190	0.0653	0.0282	0.0482
**>35**	11	28.95%							
**CA199**									
**<27**	28	73.68%	0.7577	0.9037	0.9082	0.7326	0.8458	0.6939	0.9968
**>27**	10	26.32%							
**HE4**									
**<105.1**	25	65.79%	0.2464	0.1712	0.2196	0.4011	0.0739	0.1640	0.1669
**>105.1**	13	34.21%							

## Abbreviations

UCEC: Uterine Corpus Endometrial Carcinoma
lncRNAs: Long non-coding RNAs
ceRNA: competitive endogenous RNA
MREs: miRNA response elements
miRNAs: microRNAs
mRNAs: messenger RNAs
KEGG: Kyoto Encyclopedia of Genes and Genomes
GO: Gene Ontology
FDR: False Discovery Rate
AUC: Area under curve
ROC: Receiver operating characteristics
LASSO: Least absolute shrinkage and selection operator
