# Supplementary material for: Identification of lncRNAs associated with uterine corpus endometrial cancer prognosis based on the competing endogenous RNA network
Source: Int J Med Sci. 2023 Sep 25;20(12):1600–15. doi: 10.7150/ijms.87430 (PMC10583181; doi:10.7150/ijms.87430)
Supplement: Supplementary file 1 — Supplementary tables. [file ijmsv20p1600s1.pdf]

Supplementary Table S1 Target relationship between differential lncRNAs and miRNAs

miRNA	lncRNA
**hsa-miR-101-3p**	C4orf38, SNHG3, DIRC3, HCG11
**hsa-miR-103a-3p**	C14orf64, HAR1A, UCA1, C8orf12
**hsa-miR-106a-5p**	DIO3OS, SNHG10, C14orf64, SNHG3, C8orf12, C12orf36, HCG11
**hsa-miR-106b-5p**	C14orf64, SNHG3, HCG11, C8orf12, C12orf36
**hsa-miR-107**	C14orf64, HAR1A, UCA1, C8orf12
**hsa-miR-10a-5p**	DIO3OS, C8orf51, SNHG3, DIRC3
**hsa-miR-10b-5p**	DIO3OS, C8orf51, SNHG3, DIRC3
**hsa-miR-124-3p**	DIO3OS, SNHG10, C14orf64, UCA1, C8orf12
**hsa-miR-125a-5p**	DIO3OS, C4orf38, C14orf64, DIRC3, HAR1A
**hsa-miR-125b-5p**	DIO3OS, C4orf38, C14orf64, DIRC3, HAR1A
**hsa-miR-128-3p**	C14orf64, SNHG3, C1orf133, DSCR8
**hsa-miR-129-5p**	C14orf64, SNHG3, HCG11, UCA1, DSCR8, C12orf36
**hsa-miR-130a-3p**	C14orf64
**hsa-miR-133a-3p**	DIO3OS, DIRC3
**hsa-miR-133b**	DIO3OS, DIRC3
**hsa-miR-135a-5p**	C14orf64, SNHG3, UCA1, C12orf36
**hsa-miR-135b-5p**	C14orf64, SNHG3, UCA1, C12orf36
**hsa-miR-137**	C14orf64, DSCR8
**hsa-miR-138-5p**	DIO3OS, C4orf38, UCA1, C12orf36
**hsa-miR-140-5p**	DIRC3, C12orf36
**hsa-miR-141-3p**	SNHG3, C8orf12, C12orf36
**hsa-miR-142-3p**	C4orf38, C14orf64, DIRC3
**hsa-miR-142-5p**	C4orf38, C14orf64, DIRC3
**hsa-miR-143-3p**	DIO3OS, C8orf51, C14orf64, HCG11, UCA1
**hsa-miR-144-3p**	C4orf38, C14orf64, DIRC3, HCG11
**hsa-miR-145-5p**	DIRC3, C8orf12, C12orf36
**hsa-miR-148a-3p**	C14orf64, SNHG3, DSCR8
**hsa-miR-148b-3p**	C14orf64, SNHG3, DSCR8
**hsa-miR-152-3p**	C14orf64, SNHG3, DSCR8
**hsa-miR-153-3p**	C14orf64, DSCR8
**hsa-miR-155-5p**	DIRC3, HCG11, C12orf36
**hsa-miR-15a-5p**	C14orf64, DIRC3, C1orf133, C8orf12
**hsa-miR-15b-5p**	C14orf64, DIRC3, C1orf133, C8orf12
**hsa-miR-16-5p**	C14orf64, DIRC3, C1orf133, C8orf12
**hsa-miR-17-5p**	C14orf64, SNHG3, HCG11, C8orf12, C12orf36
**hsa-miR-181a-5p**	DIO3OS, HCG11, DSCR8
**hsa-miR-181b-5p**	DIO3OS, HCG11, DSCR8
**hsa-miR-181c-5p**	DIO3OS, HCG11, DSCR8
**hsa-miR-182-5p**	SNHG10, SNHG3, UCA1, C12orf36
**hsa-miR-183-5p**	DIRC3, C12orf36

**hsa-miR-18a-5p**	DIO3OS, UCA1
**hsa-miR-195-5p**	C14orf64, DIRC3, C1orf133, C8orf12
**hsa-miR-196a-5p**	C8orf51, SNHG3, HCG11
**hsa-miR-196b-5p**	C8orf51, SNHG3, HCG11
**hsa-miR-199a-3p**	DIO3OS, SNHG9, C8orf51, C14orf64, DIRC3
**hsa-miR-199a-5p**	DIO3OS, SNHG9, C8orf51, C14orf64, DIRC3
**hsa-miR-199b-5p**	DIO3OS, SNHG9, C8orf51, C14orf64, DIRC3
**hsa-miR-19a-3p**	DIO3OS, C14orf64, SNHG3, DIRC3, C12orf36
**hsa-miR-19b-3p**	DIO3OS, C14orf64, SNHG3, DIRC3, C12orf36
**hsa-miR-200a-3p**	SNHG3, C8orf12, C12orf36
**hsa-miR-200b-3p**	DIRC3
**hsa-miR-200c-3p**	DIRC3
**hsa-miR-204-5p**	DIO3OS, DIRC3, HCG11, C1orf133, C8orf12
**hsa-miR-205-5p**	C8orf51, C4orf38, HCG11, SNHG3, DIRC3, DSCR8
**hsa-miR-206**	DIO3OS, UCA1, SNHG3, DIRC3, C12orf36
**hsa-miR-20a-5p**	C14orf64, SNHG3, HCG11, C8orf12, C12orf36
**hsa-miR-20b-5p**	C14orf64, SNHG3, HCG11, C8orf12, C12orf36
**hsa-miR-211-5p**	DIO3OS, DIRC3, HCG11, C1orf133, C8orf12
**hsa-miR-212-3p**	C12orf36
**hsa-miR-217**	DIO3OS, SNHG10, C14orf64, HCG11, C1orf133
**hsa-miR-218-5p**	DIO3OS, C8orf51, C14orf64, DIRC3, HCG11
**hsa-miR-22-3p**	C4orf38, C14orf64, HAR1A, DSCR8, C12orf36
**hsa-miR-221-3p**	DIO3OS, C14orf64, SNHG3, C12orf36
**hsa-miR-222-3p**	DIO3OS, C14orf64, SNHG3, C12orf36
**hsa-miR-223-3p**	C1orf133, C8orf12, C12orf36
**hsa-miR-23a-3p**	C14orf64, UCA1, C8orf12
**hsa-miR-23b-3p**	C14orf64, UCA1, C8orf12
**hsa-miR-24-3p**	DIO3OS, C8orf51, SNHG10, C14orf64, SNHG3, DIRC3, HCG11, HAR1A
**hsa-miR-25-3p**	C14orf64, C8orf12
**hsa-miR-26a-5p**	C4orf38, C14orf64, DIRC3, HCG11, UCA1, C12orf36
**hsa-miR-26b-5p**	C4orf38, C14orf64, DIRC3, HCG11, UCA1, C12orf36
**hsa-miR-27a-3p**	DIO3OS, HAR1A, C1orf133, DSCR8
**hsa-miR-27b-3p**	DIO3OS, HAR1A, C1orf133, DSCR8
**hsa-miR-29a-3p**	SNHG10, C14orf64, DIRC3, C8orf12, C12orf36
**hsa-miR-29b-3p**	SNHG10, C14orf64, DIRC3, C8orf12, C12orf36
**hsa-miR-29c-3p**	SNHG10, C14orf64, DIRC3, C8orf12, C12orf36
**hsa-miR-301a-3p**	C14orf64
**hsa-miR-302d-3p**	DIO3OS, SNHG10, C14orf64, SNHG3, C8orf12, C12orf36
**hsa-miR-32-5p**	C14orf64, C8orf12
**hsa-miR-33a-5p**	C14orf64, C8orf51, C12orf36
**hsa-miR-34a-5p**	DIO3OS, C8orf51, C4orf38, C14orf64
**hsa-miR-34c-5p**	DIO3OS, C8orf51, C4orf38, C14orf64
**hsa-miR-372-3p**	DIO3OS, SNHG10, C14orf64, SNHG3, C8orf12, C12orf36
**hsa-miR-373-3p**	DIO3OS, SNHG10, C14orf64, SNHG3, C8orf12, C12orf36

**hsa-miR-424-5p**	C14orf64, DIRC3, C1orf133, C8orf12
**hsa-miR-429**	DIRC3
**hsa-miR-449a**	DIO3OS, C8orf51, C4orf38, C14orf64
**hsa-miR-497-5p**	C14orf64, DIRC3, C1orf133, C8orf12
**hsa-miR-506-3p**	DIO3OS, SNHG10, C14orf64, UCA1, C8orf12
**hsa-miR-519d-3p**	C14orf64, SNHG3, HCG11, C8orf12, C12orf36
**hsa-miR-7-5p**	DIO3OS, C8orf51, DIRC3, HCG11, C12orf36
**hsa-miR-9-5p**	C14orf64, DIRC3, C1orf133, C12orf36
**hsa-miR-92a-3p**	C14orf64, C8orf12
**hsa-miR-93-5p**	DIO3OS, SNHG10, C14orf64, SNHG3, C8orf12, C12orf36, HCG11
**hsa-miR-96-5p**	SNHG10, C14orf64, DIRC3, UCA1, C12orf36
**hsa-miR-98-5p**	DIO3OS, DIRC3, HCG11, DSCR8

Supplementary Table S2 Target relationship between miRNA and differential mRNA

**miRNA**	**lncRNA**
**hsa-miR-101-3p**	TSHZ3, SOCS5, RAP2C, MYCN, MBNL1, EZH2, DUSP1, CACNB2, ATXN1
**hsa-miR-103a-3p**	ZFPM2, VCL, TSPAN5, TGFBR3, SYNJ1, SNRK, RUNX1T1, RNF38, RAP2C, RAB11FIP2, MBNL1, KIF23, FERMT2, CCNE1
**hsa-miR-106a-5p**	ZBTB4, TXNIP, SLITRK3, SAR1B, RBL2, RASSF2, PKD2, PBX3, NR4A3, NR4A2, KAT2B, FBXL5, EZH1, CRIM1, CFL2, ATXN1, ARID4A
**hsa-miR-106b-5p**	ZFPM2, ZBTB47, ZBTB4, TXNIP, TSHZ3, TNFRSF21, SMOC2, SLITRK3, SKI, SAR1B, RBL2, RAP2C, PKD2, PKD1, PBX3, NR4A3, NR4A2, NBEA, MYCN, MAP3K9, MAP3K3, KPNA2, KIF23, KAT2B, HLF, HBP1, FBXW11, FBXL5, EZH1, ELK3, DPYSL2, CRIM1, CHD9, CFL2, CALD1, ARID4A
**hsa-miR-107**	ZFPM2, VCL, TGFBR3, SYNJ1, SNRK, RUNX1T1, RNF38, RAP2C, RAB11FIP2, MBNL1, KIF23, FERMT2, CCNE1, CACNA2D1
**hsa-miR-10a-5p**	ZMYND11, TFAP2C, NR4A3, GATA6
**hsa-miR-10b-5p**	ZMYND11, TFAP2C, NR4A3, GATA6
**hsa-miR-124-3p**	ZCCHC24, VAMP3, RYR3, PALLD, NR3C2, MITF, MAPK14, ANTXR2
**hsa-miR-125a-5p**	STARD13, ARID3B
**hsa-miR-125b-5p**	TDG, STARD13, MAMDC2, LIFR, ARID3B
**hsa-miR-128-3p**	SYT1, SMARCA2, RNF38, RNF144A, PDE7B, MAPK14, CORO1C, CITED2
**hsa-miR-129-5p**	SOX4
**hsa-miR-130a-3p**	ZFPM2, RNF38, RAP2C, NPTN, MEMO1, MDFIC, MBNL1, HBP1, FOXF2, ELK3, E2F7, DPYSL2, CLIP1, CHD9, ATXN1, ADAM12, ABHD3
**hsa-miR-133a-3p**	MECOM, LHFP
**hsa-miR-133b**	SGMS2, RAP2C, MECOM, LHFP
**hsa-miR-135a-5p**	ROCK2, NR3C2, MEF2C, FOXO1, FOXN3
**hsa-miR-135b-5p**	NR3C2, FOXN3
**hsa-miR-137**	ZC3H6, TJP1, SYT1, SNRK, SLC30A4, RAP2C, PDE7A, NBEA, MSI1, MITF, MBNL2, KCNMB2, INPP5A, HLF, ESRRA, CXCL12, CHD9
**hsa-miR-138-5p**	ZMYND11, ROCK2, PDE7B, NBEA, MACF1, KLF11, FERMT2, EZH2
**hsa-miR-140-5p**	PITX2

**hsa-miR-141-3p**	ZEB2, TSHZ3, CHD9, ATXN1
**hsa-miR-142-3p**	ZEB2, KAT2B, INPP5A
**hsa-miR-142-5p**	ZFPM2
**hsa-miR-143-3p**	IGFBP5
**hsa-miR-144-3p**	ZFX, ZEB2, NR2F2, MITF, MBNL2, MBNL1, FBXW11, DMD, CXCL12, ATXN1
**hsa-miR-145-5p**	PMP22, MDFIC, FLI1, AKAP12
**hsa-miR-148a-3p**	SYNJ1, S1PR1, RNF38, PDIA3, NPTN, MITF, MAP3K9
**hsa-miR-148b-3p**	ZDHHC17, SYNJ1, S1PR1, RNF38, NPTN, MITF, MAP3K9
**hsa-miR-152-3p**	ZDHHC17, TXNIP, SYNJ1, S1PR1, RNF38, PDIA3, NPTN, MITF, MAP3K9, KLF4, E2F7, DUSP1, DNMT1, CHD9, ATXN1
**hsa-miR-153-3p**	ZFPM2, SNCA, SLCO5A1, SETD7, RYR3, ROCK2, NR3C2, NPTN, FOXO1, DMD, CITED2, ARID4A, ARF1, APC, ADD1
**hsa-miR-155-5p**	TSHZ3, RPS6KA3, FGF7
**hsa-miR-15a-5p**	USP25, TMEM55A, SYNJ1, SNRK, SESN1, RUNX1T1, RECK, RAP2C, PID1, MAP7, GRM7, FGF7, FGF2, FERMT2, E2F3, CDK17, CCNE1, BCL2L2, AKT3
**hsa-miR-15b-5p**	USP25, TMEM55A, SYNJ1, SNRK, SESN1, RUNX1T1, RECK, RAB11FIP2, PID1, MAP7, GRM7, FGF7, FGF2, FERMT2, ESRRA, E2F3, CDK17, CCNE1, CCND2, BCL2L2, AKT3
**hsa-miR-16-5p**	ZYX, USP25, TMEM55A, TGFBR3, TAB3, SYNJ1, SPRYD3, SNRK, SESN1, RUNX1T1, RSPO3, RECK, RAP2C, RAB11FIP2, PID1, MAP7, LATS2, KIF23, GRM7, GHR, FGF7, FGF2, FERMT2, ESRRA, E2F7, E2F3, CRIM1, CDK17, CCNE1, CCND2, BCL2L2, AKT3
**hsa-miR-17-5p**	ZFPM2, ZBTB4, TXNIP, TSHZ3, TNFRSF21, SOX4, SMOC2, SLITRK3, SKI, SAR1B, RBL2, RASSF2, RAPGEF4, RAP2C, RAB30, PKD2, PKD1, PBX3, NR4A3, NR4A2, NBEA, MYCN, MAP3K9, MAP3K3, LHX6, KPNA2, KIF23, KCNMA1, KAT2B, HLF, HBP1, FBXW11, FBXL5, ELK3, DPYSL2, CRIM1, CHD9, CFL2, CALD1, BNC2, ATXN1, ARID4A, AKT3
**hsa-miR-181a-5p**	NR4A3, ATXN1
**hsa-miR-181b-5p**	ATXN1
**hsa-miR-181c-5p**	NR4A3, ATXN1
**hsa-miR-182-5p**	VCL, VAMP3, PRKACB, PALLD, MITF, MBNL2, JAZF1, FOXN3, FOXF2, FBXW11, EPAS1, EBF3, CITED2
**hsa-miR-183-5p**	ITGB1
**hsa-miR-18a-5p**	CRIM1, ATXN1
**hsa-miR-195-5p**	USP25, TMEM55A, TGFBR3, TAB3, SYNJ1, SPRYD3, SNRK, SESN1, RUNX1T1, RSPO3, RECK, RAP2C, RAB11FIP2, PID1, MAP7, LATS2, GRM7, GHR, FGF7, FGF2, FERMT2, ESRRA, E2F7, E2F3, CRIM1, CDK17, CCNE1, CCND2, BCL2L2, AKT3
**hsa-miR-196a-5p**	ZMYND11, HOXB7, GATA6
**hsa-miR-196b-5p**	ZMYND11, HOXB7
**hsa-miR-199a-3p**	CITED2, CDK17
**hsa-miR-199a-5p**	SLC24A3
**hsa-miR-199b-5p**	SLC24A3

**hsa-miR-19a-3p**	ZMYND11, ZFPM2, UBL3, TSHZ3, SYT1, SOX4, SOCS1, SMOC2, SMARCA2, SLC24A3, S1PR1, RNF38, RAPGEF4, RAP2C, RAP1A, RAB8B, PRICKLE2, PPP1R12A, NBEA, NAV3, MEMO1, MBNL2, MBNL1, KCNA4, IVNS1ABP, IMPDH1, HBP1, FOXF2, ELK3, E2F8, CLIP1, BTG1, BNC2, ATXN1
**hsa-miR-19b-3p**	ZMYND11, ZFPM2, UBL3, TSHZ3, SYT1, SOX4, SOCS1, SMOC2, SMARCA2, SLC24A3, S1PR1, RNF38, RAPGEF4, RAP2C, RAP1A, RAB8B, PRICKLE2, PPP1R12A, NBEA, NAV3, MEMO1, MBNL1, KCNA4, IVNS1ABP, IMPDH1, HBP1, FOXF2, ELK3, E2F8, CLIP1, ATXN1, ACTN1
**hsa-miR-200a-3p**	ZEB2, TSHZ3, PITX2, MBNL1, H2AFZ, GATA6, E2F3, DNAJC13, CHD9, ATXN1
**hsa-miR-200b-3p**	ZFPM2, SEC23A, FEZ2, EPS8, DUSP1, CDK17
**hsa-miR-200c-3p**	ZEB2, ZEB1, SEC23A, FEZ2, EPS8, DUSP1, CDK17
**hsa-miR-204-5p**	ZCCHC24, RAP2C, NR3C1
**hsa-miR-205-5p**	RBM47
**hsa-miR-206**	TPM3, TIMP3, TAGLN2, SYT1, STC2, PRKACB, NR4A2, HSP90B1, HDAC4, CPEB1, CORO1C, AKAP11, ADPGK
**hsa-miR-20a-5p**	ZFPM2, TSHZ3, SLITRK3, SAR1B, RBL2, RAP2C, PBX3, NR4A3, NR4A2, NBEA, MAP3K9, KAT2B, HBP1, FBXW11, FBXL5, ELK3, CRIM1, CHD9, CFL2, CALD1, ARID4A
**hsa-miR-20b-5p**	ZBTB4, TXNIP, TNFRSF21, SAR1B, RBL2, PKD2, NR4A3, NBEA, MAP3K9, FBXL5, EZH1, CHD9, FL2, ARID4A
**hsa-miR-211-5p**	NR3C1
**hsa-miR-212-3p**	H2AFZ, CFL2
**hsa-miR-217**	UBL3, ANLN
**hsa-miR-218-5p**	ZEB2, SLCO5A1, RNF38, RAB8B, PRKG1, NMT2, NAV3, MAGI2, HLF, HAPLN1, EBF3, DNAJC13, ACTN1
**hsa-miR-219a-5p**	TSC22D2, SNRK
**hsa-miR-22-3p**	TAGLN, SNRK, NR3C1, MAPK14, ERBB3, CPEB1, CHD9, AKT3
**hsa-miR-221-3p**	NAP1L5, MAPK10
**hsa-miR-222-3p**	NAP1L5
**hsa-miR-223-3p**	HLF, CRIM1, CDK17
**hsa-miR-23a-3p**	PPIF, PDE7A, NACC2, CXCL12
**hsa-miR-23b-3p**	RUNX1T1, PPIF, PDE7A, NEK6, NACC2, CXCL12
**hsa-miR-24-3p**	RAP2C
**hsa-miR-25-3p**	ZEB2, SYNJ1, SOX4, S1PR1, ROBO2, RNF38, RBM47, RAB23, PPP1R12A, NPTN, MYCBP2, LATS2, KLF4, KAT2B, IQGAP2, GRHL1, GATA6, FZD10, FRY, FOXG1, DPP10, ADM
**hsa-miR-26a-5p**	YWHAE, STRBP, RAP2C, PPP3CB
**hsa-miR-26b-5p**	STRBP, RAP2C, E2F7
**hsa-miR-27a-3p**	TRIM23, RUNX1, RPN2, PPIF, NR2F6
**hsa-miR-27b-3p**	TRIM23, SLC25A25, PDS5B, PDE7B, NR2F6, NEDD4, EBF3, E2F7, DNAJC13
**hsa-miR-29a-3p**	TDG, RAB30, PMP22, NASP, IFI30, HBP1, FEM1B
**hsa-miR-29b-3p**	TDG, PMP22, NAV3, HBP1, FEM1B

**hsa-miR-29c-3p**	TDG, RAB30, RAB15, PMP22, NAV3, IFI30, HBP1, FERMT2, FEM1B, DNMT3A, COL7A1, ATXN1
**hsa-miR-301a-3p**	ZFPM2, S1PR1, RNF38, RAPGEF4, RAP2C, NPTN, MEMO1, MDFIC, MBNL1, HBP1, FOXF2, ELK3, E2F7, DPYSL2, CLIP1, CHD9, ATXN1, ADAM12
**hsa-miR-302d-3p**	EDNRB
**hsa-miR-32-5p**	ZEB2, TEAD1, SYNJ1, SOX4, S1PR1, RNF38, RBM47, RAB23, PPP1R12A, NPTN, MYCBP2, LATS2, KLF4, KAT2B, ITPR1, ITGAV, IQGAP2, HAND2, GRHL1, GATA6, FZD10, FRY, FOXN3, FOXG1, EPS8, DPP10, DMXL1
**hsa-miR-33a-5p**	SNRK
**hsa-miR-34a-5p**	ZDHHC17, SYT1, NAV3
**hsa-miR-34c-5p**	SYT1, NAV3
**hsa-miR-372-3p**	TXNIP, R4A3, LATS2, KAT2B
**hsa-miR-373-3p**	LATS2, EDNRB
**hsa-miR-424-5p**	PID1
**hsa-miR-429**	ZEB1, SEC23A
**hsa-miR-449a**	SYT1
**hsa-miR-497-5p**	CCNE1
**hsa-miR-506-3p**	VAMP3
**hsa-miR-519d-3p**	TXNIP, RBL2, NBEA
**hsa-miR-7-5p**	VDAC1, SNCA, NR4A3, KLF4, FOXN3, ATXN1, ARID4A
**hsa-miR-9-5p**	VCL, VAV3, TSC22D2, SYNJ1, SOCS5, SNRK, RANBP17, PDK4, NEDD4, MBNL1, MAP7, MAP3K3, KCTD12, KCNMB2, GOT1, FOXG1, DIO2, CRIM1, ATXN1
**hsa-miR-92a-3p**	TEAD1, SYNJ1, RBM47, RAB23, NR4A3, NPTN, MYCBP2, LATS2, KLF4, KAT2B, GRHL1, GATA6, FRY, DPP10
**hsa-miR-93-5p**	ZFPM2, TXNIP, TSHZ3, SLITRK3, SAR1B, RBL2, PBX3, NR4A3, NR4A2, NBEA, MYCN, MAP3K3, LHX6, KAT2B, HBP1, FBXL5, CRIM1, CHD9, CFL2, ARID4A
**hsa-miR-96-5p**	ZDHHC17, TTYH3, TACC1, SH3BP5, RAB23, PRKAR1A, MITF, ITPR1, INPP5A, HBEGF, GRHL2, FOXO1, FBXW11, EBF3, ATXN1
**hsa-miR-98-5p**	TSC22D2, PBX3, MYCN, KLF9, FRAS1, DUSP1, DMD, DLC1, CCNJ, CCND2, ARID3B, ADAMTS5

Supplementary Table S3 mRNA-miRNA-lncRNA interactions with mRNAs of oncogenes, tumor suppressor genes and apoptosis related genes

mRNA	miRNA	lncRNA
**Tumor suppressor genes**		
**APC**	hsa-miR-153-3p	C14orf64, DSCR8
**BTG1**	hsa-miR-19a-3p	C14orf64, SNHG3, C12orf36, DIRC3, DIO3OS
**LATS2**	hsa-miR-372-3p, hsa-miR-25-3p, hsa-miR-16-5p, hsa-miR-195-5p, hsa-miR-373-3p, hsa-miR-32-5p, hsa-miR-92a-3p	SNHG3, SNHG10, C12orf36, DIO3OS, C14orf64, C8orf12, DIRC3, C1orf133
**ROBO2**	hsa-miR-25-3p	C14orf64, C8orf12
**RUNX1**	hsa-miR-27a-3p	HAR1A, DSCR8, DIO3OS, C1orf133
**SOCS1**	hsa-miR-19b-3p, hsa-miR-19a-3p	C14orf64, DIO3OS, C12orf36, DIRC3, SNHG3
**TPM3**	hsa-miR-206	SNHG3, UCA1, C12orf36, DIO3OS, DIRC3
**YWHAE**	hsa-miR-26a-5p	C4orf38, HCG11, UCA1, C14orf64, DIRC3, C12orf36
**Oncogenes**		
**AKT3**	hsa-miR-17-5p, hsa-miR-16-5p, hsa-miR-22-3p, hsa-miR-15a-5p, hsa-miR-15b-5p, hsa-miR-195-5p	C8orf12, C14orf64, C12orf36, SNHG3, HCG11, DIRC3, C1orf133, C4orf38, DSCR8, HAR1A
**CCND2**	hsa-miR-15b-5p, HSA-MIR-98-5P, hsa-miR-195-5p, hsa-miR-16-5p	DIRC3, C1orf133, C8orf12, C14orf64
**CCNE1**	hsa-miR-15a-5p, hsa-miR-195-5p, hsa-miR-16-5p, hsa-miR-103a-3p, hsa-miR-497-5p, hsa-miR-15b-5p, hsa-miR-107	C8orf12, C14orf64, DIRC3, C1orf133, HAR1A, UCA1
**DNMT1**	hsa-miR-152-3p	DSCR8, C14orf64, SNHG3
**ERBB3**	hsa-miR-22-3p	C4orf38, DSCR8, HAR1A, C14orf64, C12orf36
**EZH2**	hsa-miR-138-5p, hsa-miR-101-3p	C4orf38, DIO3OS, C12orf36, UCA1, HCG11, SNHG3, DIRC3

**FLI1**	hsa-miR-145-5p	DIRC3, C12orf36, C8orf12
**HLF**	hsa-miR-137, hsa-miR-218-5p, hsa-miR-223-3p, hsa-miR-17-5p, hsa-miR-106b-5p	C14orf64, DSCR8, DIO3OS, DIRC3, HCG11, C8orf51, C1orf133, C12orf36, C8orf12, SNHG3
**KLF4**	hsa-miR-92a-3p, hsa-miR-32-5p, hsa-miR-25-3p, hsa-miR-7-5p, hsa-miR-152-3p	C14orf64, C8orf12, DIO3OS, C12orf36, DIRC3, HCG11, C8orf51, DSCR8, SNHG3
**MECOM**	hsa-miR-133b, hsa-miR-133a-3p	DIRC3, DIO3OS
**MITF**	hsa-miR-152-3p, hsa-miR-148b-3p, hsa-miR-144-3p, hsa-miR-124-3p, hsa-miR-148a-3p, hsa-miR-137, hsa-miR-182-5p, HSA-MIR-96-5P	DSCR8, C14orf64, SNHG3, C4orf38, DIRC3, HCG11, DIO3OS, SNHG10, UCA1, C8orf12, C12orf36
**MYCN**	hsa-miR-17-5p, hsa-miR-106b-5p, hsa-miR-93-5p, hsa-miR-101-3p, HSA-MIR-98-5P	C8orf12, C14orf64, C12orf36, SNHG3, HCG11, SNHG10, DIO3OS, DIRC3, C4orf38
**NR4A3**	hsa-miR-17-5p, hsa-miR-181a-5p, hsa-miR-106b-5p, hsa-miR-93-5p, hsa-miR-20a-5p, hsa-miR-181c-5p, hsa-miR-7-5p, hsa-miR-92a-3p, hsa-miR-20b-5p, hsa-miR-10a-5p, hsa-miR-10b-5p, hsa-miR-106a-5p	C8orf12, C14orf64, C12orf36, SNHG3, HCG11, DSCR8, DIO3OS, SNHG10, DIRC3, C8orf51
**RSPO3**	hsa-miR-195-5p, hsa-miR-16-5p	C14orf64, C8orf12, C1orf133, DIRC3
**SKI**	hsa-miR-17-5p, hsa-miR-106b-5p	C8orf12, C14orf64, C12orf36, SNHG3, HCG11
**ZEB1**	hsa-miR-429, hsa-miR-200c-3p	DIRC3
**Apoptosis-related genes**		
**ADD1**	hsa-miR-153-3p	C14orf64, DSCR8
**AKT3**	hsa-miR-17-5p, hsa-miR-16-5p, hsa-miR-22-3p, hsa-miR-15a-5p, hsa-miR-15b-5p, hsa-miR-195-5p	C8orf12, C14orf64, C12orf36, SNHG3, HCG11, DIRC3, C1orf133, C4orf38, DSCR8, HAR1A
**APC**	hsa-miR-153-3p	C14orf64, DSCR8
**TJP1**	hsa-miR-137	C14orf64, DSCR8
**YWHAE**	hsa-miR-26a-5p	C4orf38, HCG11, UCA1, C14orf64, DIRC3, C12orf36
